# A novel signature combing cuproptosis- and ferroptosis-related genes in sepsis-induced cardiomyopathy

**DOI:** 10.3389/fgene.2023.1170737

**Published:** 2023-03-23

**Authors:** Juanjuan Song, Kairui Ren, Dexin Zhang, Xinpeng Lv, Lin Sun, Ying Deng, Huadong Zhu

**Affiliations:** ^1^ Department of Emergency, The Second Affiliated Hospital of Harbin Medical University, Harbin, China; ^2^ Department of Emergency, Peking Union Medical College Hospital, Chinese Academy of Medical Science, Beijing, China

**Keywords:** cuproptosis, ferroptosis, sepsis-induced cardiomyopathy, signature, candidates

## Abstract

**Objective:** Cardiac dysfunction caused by sepsis, usually termed sepsis-induced cardiomyopathy (SIC), is one of the most serious complications of sepsis, and ferroptosis can play a key role in this disease. In this study, we identified key cuproptosis- and ferroptosis-related genes involved in SIC and further explored drug candidates for the treatment of SIC.

**Methods:** The GSE79962 gene expression profile of SIC patients was downloaded from the Gene Expression Omnibus database (GEO). The data was used to identify differentially expressed genes (DEGs) and to perform weighted correlation network analysis (WGCNA). Furthermore, Gene Ontology (GO) and the Kyoto Encyclopedia of Genes and Genomes (KEGG) analyses were conducted. Then, gene set enrichment analysis (GSEA) was applied to further analyze pathway regulation, with an adjusted *p*-value <0.05 and a false discovery rate (FDR) <0.25. Ferroptosis-related genes were obtained from the FerrDb V2 database, and cuproptosis-related genes were obtained from the literature. We constructed a novel signature (CRF) by combing cuproptosis-related genes with ferroptosis-related genes using the STRING website. The SIC hub genes were obtained by overlapping DEGs, WGCNA-based hub genes and CRF genes, and receiver operating characteristic (ROC) curve analysis was used to determine the diagnostic value of hub genes. A transcription factor-microRNA-hub gene network was also constructed based on the miRnet database. Finally, potential therapeutic compounds for SIC were predicted based on the Drug Gene Interaction Database.

**Results:** We identified 173 DEGs in SIC patients. Four hub modules and 411 hub genes were identified by WGCNA. A total of 144 genes were found in the CRF. Then, POR, SLC7A5 and STAT3 were identified as intersecting hub genes and their diagnostic values were confirmed with ROC curves. Drug screening identified 15 candidates for SIC treatment.

**Conclusion:** We revealed that the cuproptosis- and ferroptosis-related genes, POR, SLC7A5 and STAT3, were significantly correlated with SIC and we also predicted therapeutic drugs for these targets. The findings from this study will make contributions to the development of treatments for SIC.

## Introduction

Sepsis is a dysregulated host response to infection that can cause life-threatening organ dysfunction (Singer et al., 2016). It is a leading cause of mortality and critical illness worldwide. A recent study estimated that the number of sepsis cases and deaths is twice as high as previously thought (Rudd et al., 2020). Sepsis-induced cardiomyopathy (SIC) is a common and well-elucidated complication of sepsis and is associated with higher mortality rates in patients with sepsis (Hanumanthu et al., 2021). Myocardial dysfunction is characterized by cellular abnormalities, circulating mediators and instrumental parameters. However, the lack of a consensus definition and uncertainties of the pathophysiology of SIC make it difficult to identify and validate biomarkers of the disease. In Addition, the cytokine storm also makes it difficult to identify cytokine biomarkers of SIC. Bioinformatic analyses have the potential to decipher these complex signals. Tumor necrosis factor, Jak-signal transducer and activator of transcription (STAT), and hypoxia-inducible transcription factor-1, and their interactions are increasingly recognized as main factors in sepsis cardiomyopathy (Chen et al., 2020).

Ferroptosis is a newly identified iron-dependent form of cell death that is different from other forms of cell death (Yan et al., 2021) and is involved in the development of cardiomyopathy. Downregulating HO-1 expression and iron concentration can reduce ferroptosis, thereby attenuating myocardial cell injury in sepsis (Wang et al., 2020). Ferritinophagy-mediated ferroptosis is a critical mechanism contributing to sepsis-induced cardiac injury (Ning et al., 2020) and targeting ferroptosis in cardiomyocytes may be a therapeutic strategy for preventing sepsis. We therefore aimed to use bioinformatic technology to quickly identify ferroptosis-related genes in SIC. This information can then be used for the early diagnosis of SIC and the development of new treatments of the disease.

Similar to iron, copper is also an essential micronutrient. Cells exhibit cytotoxicity when the intracellular concentration of copper ions exceeds the homeostatic threshold. Copper induces cell death by targeting lipoylated TCA cycle proteins. This leads to the aggregation of fatty acylated proteins and the loss of iron-sulfur cluster proteins, which in turn triggers proteotoxic stress and ultimately cell death (Tsvetkov et al., 2022). Cuproptosis is associated with various disease conditions, including Wilson’s disease, neurodegenerative diseases, cancer (Li et al., 2022) and heart diseases (Chen et al., 2022). Copper levels are also closely related to the morbidity and mortality of cardiovascular diseases (Cui et al., 2022; Liu and Miao, 2022).

Cuproptosis- and ferroptosis-related regulatory mechanisms are expected to be novel targets for SIC treatment. However, whether cuproptosis-related genes combined with ferroptosis-related genes can be used for diagnosis and to predict responses to immunotherapy and drug sensitivity in SIC have not been addressed. This study analyzed the difference in gene expression between non-failing hearts and SIC hearts in GEO database, and also performed WGCNA. Then, the function of DEGs was determined using GO and KEGG analysis. In addition, GSEA was applied to further analyze pathway regulation. In addition, we constructed a novel signature (CRF) by combing cuproptosis-related genes with ferroptosis-related genes using the STRING website for predicting diagnosis. Besides, SIC hub genes were obtained by overlapping DEGs, WGCNA-based hub genes and CRF genes, and receiver operating characteristic (ROC) curve analysis was used to determine the diagnostic value of hub genes. A transcription factor-microRNA-hub gene network was also constructed based on the miRnet database. Finally, potential therapeutic compounds for SIC were predicted based on the Drug Gene Interaction Database, which provided a theoretical basis for clinical treatment of SIC.

## Materials and methods

### Data resource

The GSE79962 gene expression profile was downloaded from the GEO database (http://www.ncbi.nih.gov/geo) using the GEOquery package of R software (version 4.2.1). The chip platform for GSE79962 was GPL6244 (Affymetrix Human Gene 1.0 ST Array), which consists of 20 SIC human heart tissue samples and 11 healthy human heart tissue samples. The ferroptosis-related genes were obtained from the GeneCards database (https://www.genecards.org/). All data are publicly available.

### Identification of differentially expressed genes

Raw data were downloaded as MINiML files from the Gene Expression Omnibus (GEO) database. Probes were converted to gene symbols according to the platform annotation information of the normalized data. Probes with more than one gene were eliminated, and the average of genes corresponding to more than one probe was calculated. The limma package in R software (version 4.2.1) was used to study differentially expressed genes. The adjusted *p*-value was determined to correct the false positive results in the GEO datasets. “Adjusted *p* < 0.05 and log(fold change) > 1 or log(fold change) < −1” were defined as the threshold for the differential expression of genes. The data for the listed DEGs were processed, and heatmaps and volcano plots were drawn using ComplexHeatmap and ggplot2 R packages.

### Functional and pathway enrichment analysis

To further confirm the underlying function of potential targets, the data were analyzed by functional enrichment. Gene Ontology (GO) is a widely used tool for annotating genes with functions, especially molecular function (MF), biological pathways (BP), and cellular components (CC). Kyoto Encyclopedia of Genes and Genomes (KEGG) enrichment analysis is a practical resource for studying gene functions and associated high-level genome functional information. To better understand mRNA involvement in pathogenesis, ClusterProfiler package (version: 3.18.0) in R was employed to analyze the GO function of potential targets and enrichment in the KEGG pathway. The R software package, pheatmap, was used to draw heatmaps. Then, gene set enrichment analysis (GSEA) was applied for further analysis of pathway regulation. We used KEGG rest API (https://www.kegg.jp/kegg/rest/keggapi.html) to obtain the latest gene annotation. Enrichment analysis was performed using the R package, clusterProfiler (version 3.14.3). In this analysis, the minimum gene set was 5 and the maximum gene set was 5,000. A *p*-value <0.05, false discovery rate (FDR) <0.25 and |NES| >1 indicated a significantly enriched term.

### Weighted gene co-expression network analysis (WGCNA)

Human SIC heart and healthy heart tissue samples from the GSE79962 dataset were analyzed by the WGCNA R 1.70-3 package. First, Pearson’s correlation matrices and the average linkage method were both performed for all pair-wise genes. Then, a weighted adjacency matrix was constructed using a power function, *β* was a soft-thresholding parameter that could emphasize strong correlations between genes and penalize weak correlations. Meanwhile, a soft threshold was reasonably selected as the degree of scale independence reached 0.8. After choosing the power of *β* = 5, the adjacency was transformed into a topological overlap matrix (TOM), which could measure the network connectivity of a gene defined as the sum of its adjacency with all other genes for network Gene ration, and the corresponding dissimilarity (1-TOM) was calculated. To classify genes with similar expression profiles into gene modules, average linkage hierarchical clustering was conducted according to the TOM-based dissimilarity measure with a minimum size (gene group) of 30 for the genes dendrogram. The modules that correlated the most with the clinical traits were identified as SIC-related modules. All functions of hub genes with gene significance (GS) >0.2 and module membership (MM) >0.8 were analyzed by GO enrichment.

### Construction and validation of a cuproptosis- and ferroptosis-related gene signature

First, we obtained ferroptosis-related genes were from the FerrDb V2 database (http://www.zhounan.org/ferrdb/current/). Then, cuproptosis-related genes were obtained from the literature. The obtained cuproptosis-related and ferroptosis-related genes were inputed into the STRING website (https://string-db.org/), and the minimum required interaction score was set to 0.9 to obtain iron genes related to copper genes. Thus, we constructed a novel signature (CRF) by combing cuproptosis-related genes with ferroptosis-related genes.

### Intersection genes and venn analysis

A Venn diagram drawing tool (http://bioinformatics.psb.ugent.be/webtools/Venn/) was used to generate Venn diagrams of DEGs, WGCNA-based hub genes and CRF genes. Intersection genes were included in subsequent analyses.

### Identification of hub genes based on receiver operating characteristic (ROC) curve analysis

The diagnostic values of intersection genes for SIC were detected by ROC curve and area under the ROC curve (AUC) analysis using the pROC R 1.17.0.1 package.

### Construction of a transcription factor (TF)-microRNA-hub gene network

microRNAs (miRNAs) and TFs related to intersection genes were screened for based on the miRNet2/0 online database (https://www.mirnet.ca/). TFs and miRNAs related to intersection genes were identified and added to the network using Cytoscape software (version 3.8.2).

### Screening the drug-gene interaction database (DGIdb) for potential therapeutic drugs for SIC

DGIdb (https://www.dgidb.org/) was used as a drug–gene interaction database to screen for drug–gene interactions and information from papers, databases, and web resources. Therapeutic drugs for intersection genes were identified based on the DGIdb.

## Results

### Differential gene expression analysis

**FIGURE 1:**
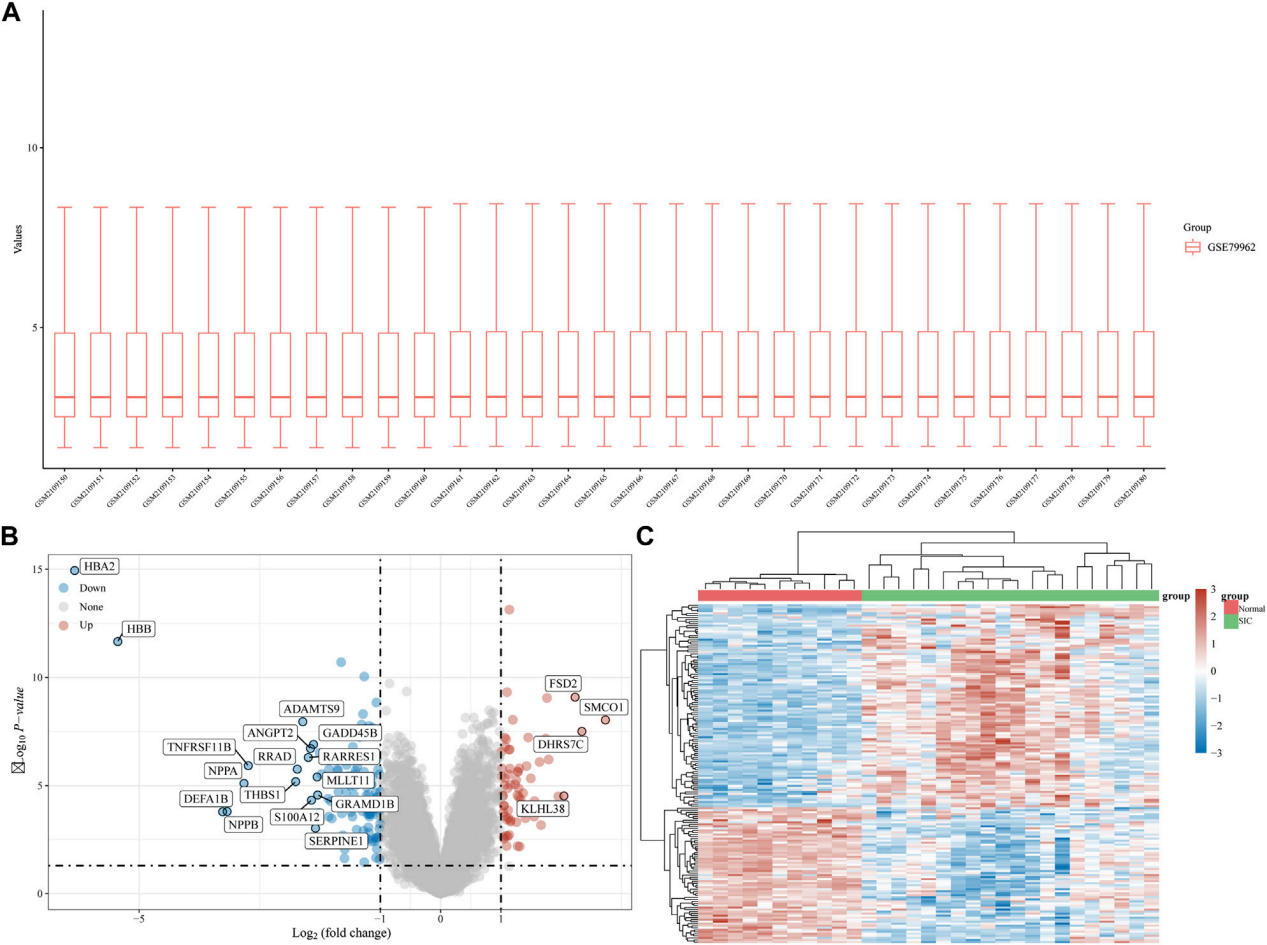
DEG analysis. **(A)** Boxplot diagram of the DEGs in the GSE79962 dataset. **(B)** Volcano plot of the DEGs in the GSE79962 dataset. **(C)** Heatmap of the DEGs in the GSE79962 dataset.

The gene expression dataset, GSE79962, contained data from 20 SIC samples, and 11 normal myocardial tissue samples. As shown in Figure 1A, data normalization and cross comparability were assessed. Using the limma package in R software (version 4.2.1) for differential expression analysis, with adjusted *p* < 0.05 and |log2 FC| >1 as filtering conditions, we found that 173 genes were differentially expressed in the myocardial tissue of patients with SIC cardiomyopathy compared with normal myocardial tissue. Sixty-seven DEGs were upregulated and 106 were downregulated. The ferroptosis-related genes (NOX4, HMOX1, POR, SAT1, etc.) were highly expressed in sepsis-induced cardiomyopathy model. Recent studies showed that NOX4 was characterized in the cardiovascular system, HMOX1 can be induced by sepsis, and POR was selected as the central gene of SIC. The above results are consistent with our research. Clustering analysis of these DEGs was performed, as shown in a volcano plot (Figure 1B). The heatmap for the dataset indicated better clustering of samples and higher confidence (Figure 1C).

### Functional pathway enrichment analysis of DEGs in SIC

**FIGURE 2:**
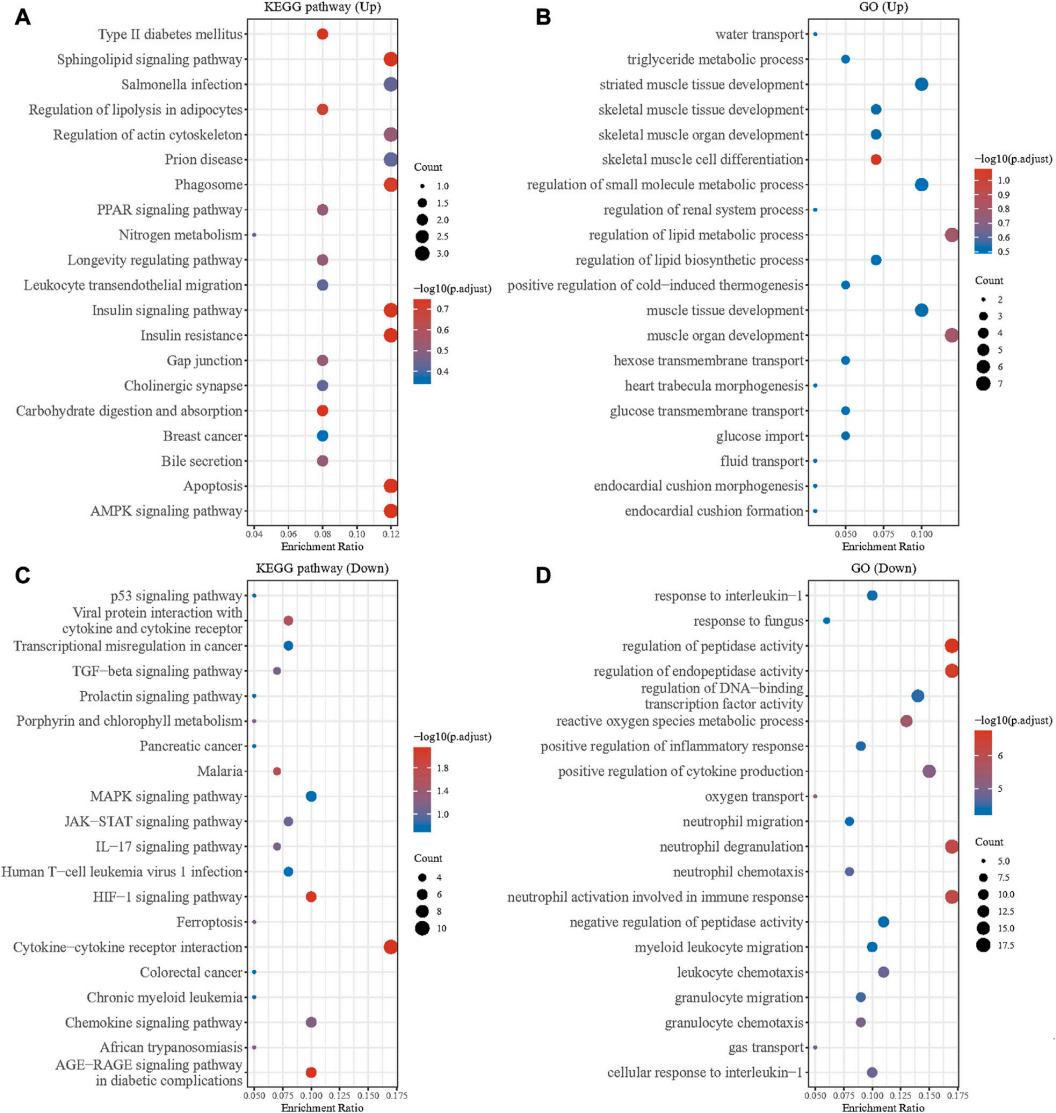
GO and KEGG enrichment analysis of DEGs. **(A)** GO enrichment analysis of the upregulated DEGs. **(B)** KEGG enrichment analysis of the upregulated DEGs. **(C)** GO enrichment analysis of the downregulated DEGs. **(D)** KEGG enrichment analysis of the downregulated DEGs.

We performed KEGG and GO enrichment analyses on the up and downregulated DEGs in the GSE79962 dataset. The results showed that there were significant differences among the functions of DEGs. The upregulated DEGs were mainly enriched in pathways of muscle tissue development, regulation of small molecule metabolic process, regulation of actin cytoskeleton, leukocyte transendothelial migration, and AMPK signaling pathway (Figures 2A, B). The downregulated DEGs were mainly enriched in pathways of response to interleukin 1, regulation of peptidase activity, positive regulation of cytokine production, neutrophil activation involved in immune response, MAPK signaling pathway, JAK-STAT signaling pathway, and cytokine-cytokine receptor interaction (Figures 2C, D). As we all know, AMPK has a strong regulatory effect on cellular energy balance, metabolic homeostasis, inflammatory response, oxidative stress and myocardial cell survival, and is closely related to the pathogenesis of septic cardiomyopathy. MAPK signaling pathway and JAK/STAT signaling is an important pathway for the signal transduction of several key cytokines in the pathogenesis of sepsis, which can transcribe and modulate the host immune response. p38-MAPK in the MAPK family is involved in SIC signaling and apoptosis mechanism. Application of clinically used JAK/STAT inhibitors, tofacitinib and baricitinib, fully prevented IFNγ-induced cardiomyopathy, confirming the critical roles of this signaling pathway in inflammatory cardiac disease. Our results are consistent with previous studies.

**FIGURE 3:**
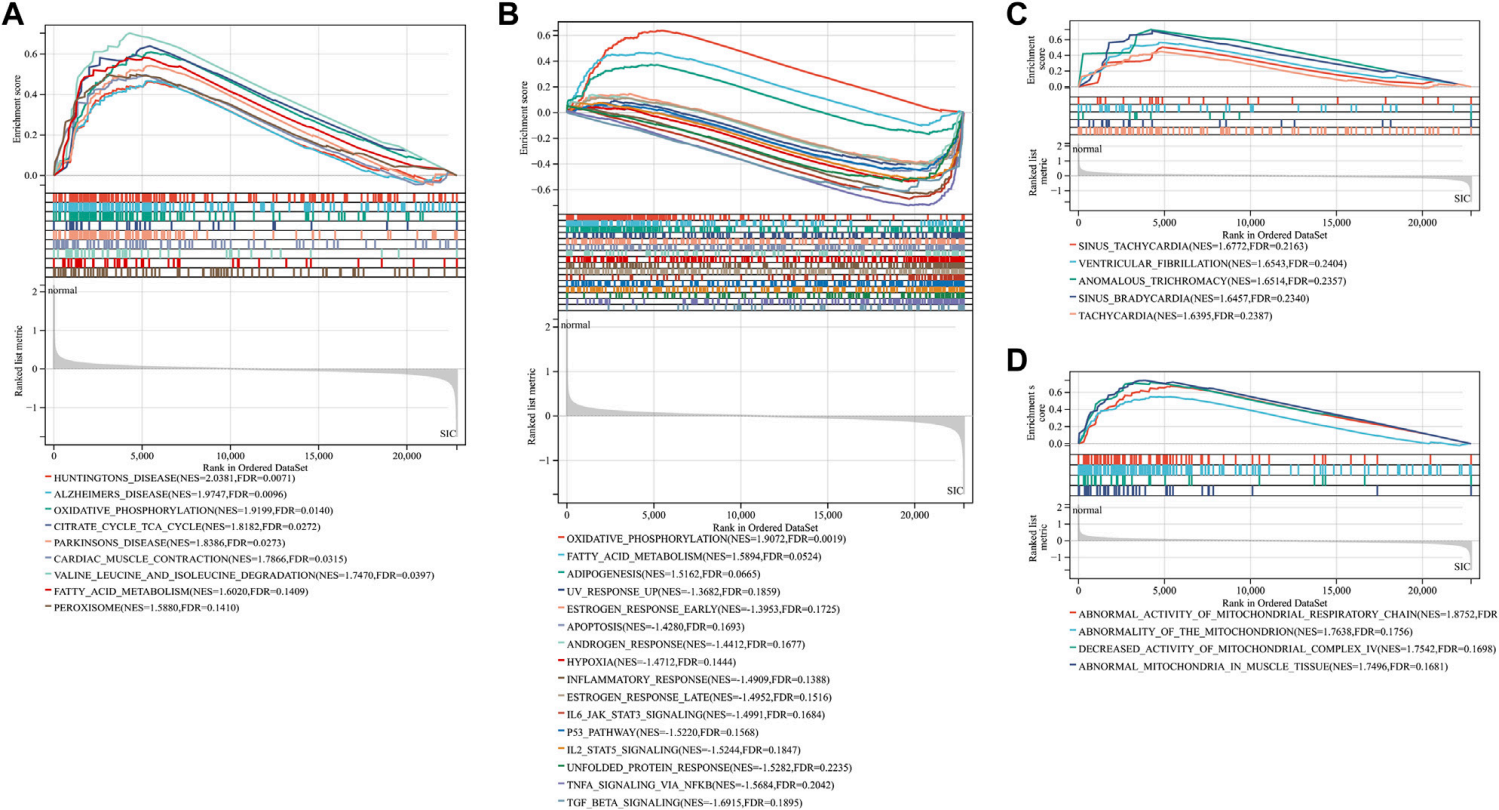
Gene set enrichment analysis (GSEA) for GSE79962. **(A)** KEGG **(B)** hallmark gene sets **(C)** arrhythmia **(D)** mitochondrion.

GSEA showed that compared with control samples, the identified KEGG pathways were Huntington’s disease, Alzheimer’s disease, oxidative phosphorylation, citrate cycle TCA cycle, Parkinson’s disease, cardiac muscle contraction, valine leucine and isoleucine degradation, fatty acid metabolism and peroxisome (Figure 3A). The identified hallmark gene sets were oxidative phosphorylation, fatty acid metabolism, adipogenesis, UV response up, estrogen response early, apoptosis, androgen response, hypoxia, inflammatory response, estrogen response late, IL6-JAK-STAT3 signaling, P53 pathway, IL2-STAT5 signaling, unfolded protein response, TNFα signaling *via* NF-κB, and TGFβ signaling (Figure 3B). The human phenotype ontologies identified were arrhythmia and mitochondrion (Figures 3C, D).

### Hub modules and genes identified by WGCNA

**FIGURE 4:**
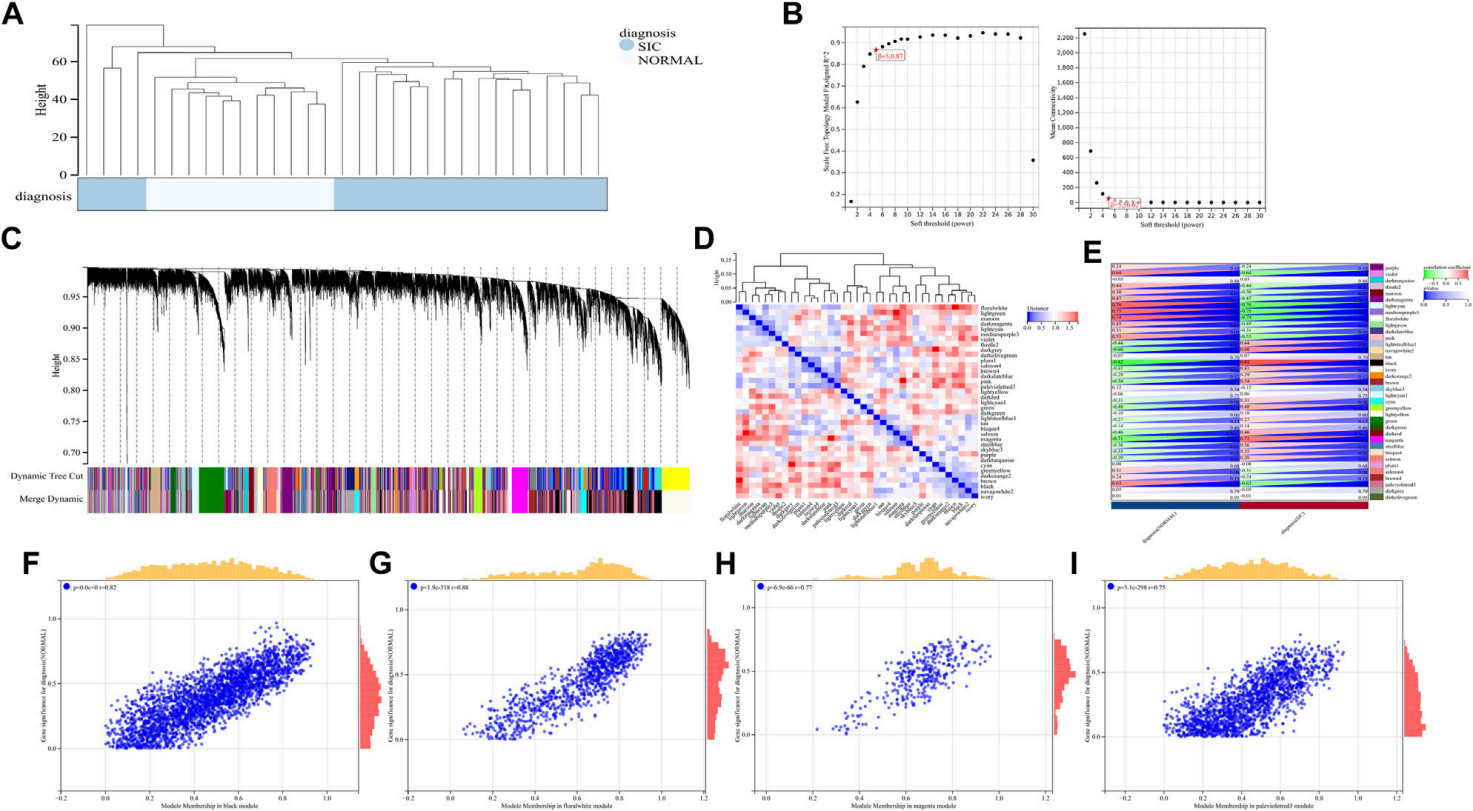
**(A–I)** WGCNA analysis of GSE79962.

A total of 22,828 genes were derived from the 31 samples of the GSE79962 dataset. These genes were used to construct a co-expression network. The cluster analysis results of the samples are shown in Figure 4. Clustering trees for each dataset were established and no outliers were found (Figure 4A). Soft threshold was reasonably selected as the degree of scale independence reached 0.8. The scale-free fit index and mean connectivity were calculated and the power of *β* = 5 (scale free R2 = 0.87) was selected (Figure 4B). The minimum number of genes per module was set to 30 according to the criteria of the dynamic tree-cutting algorithm. The final 37 transcriptional modules represented by different colors were identified (Figure 4C). The adjacencies of modules in the network are shown in Figure 4D. To correlate the modules with sample information, we analyzed the data according to the heatmap of module-clinical trait correlations, thereby correlating data for the clinical traits (Figure 4E). The black, floral white, magenta, and pale violet red 3 modules, which were identified as the hub modules associated with clinical traits, were used to explore the correlation between module membership (MM) and gene significance (GS) to identify the hub genes in SIC (Figures 4F–I). Furthermore, we demonstrated 411 hub genes were respectively identified from the above four modules with MM >0.8 and GS >0.2.

### Selection of intersection hub genes and their functions in SIC

**FIGURE 5:**
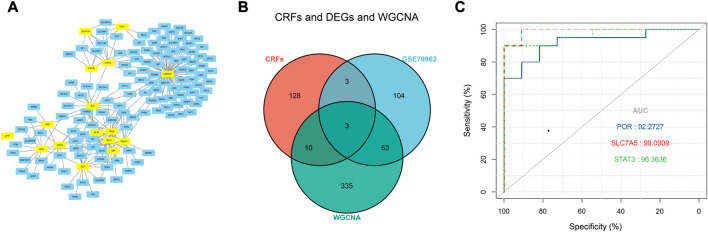
**(A)** The correlation between cuproptosis-related genes and ferroptosis-related genes (CRFs). **(B)** Venn diagram of CRFs related DEGs. **(C)** Quantification of ROC curves values of AUC for POR, SLC7A5, STAT3.

**FIGURE 6:**
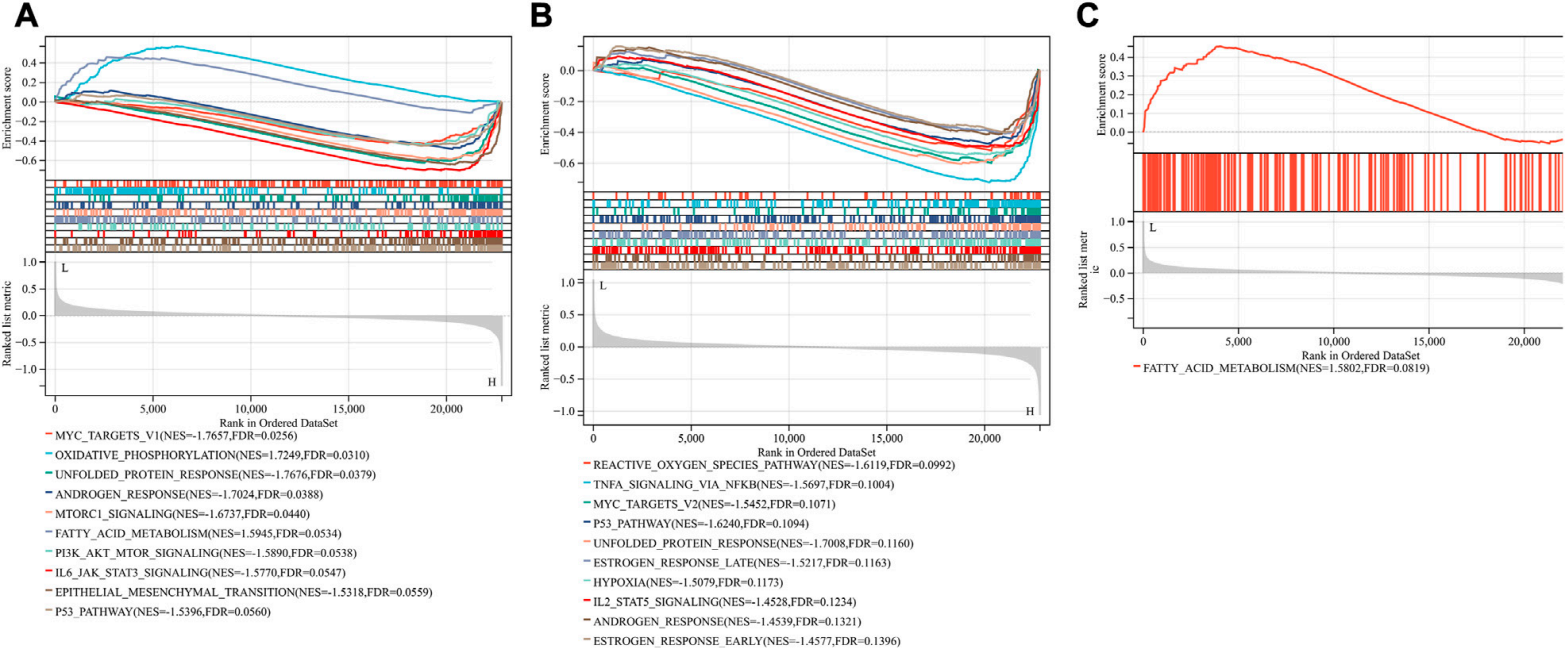
Gene set enrichment analysis (GSEA). The pathway related to three genes **(A)** POR **(B)** SLC7A5 **(C)** STAT3.

Based on the FerrDb V2 database, we obtained 612 ferroptosis-related genes. We also identified 16 cuproptosis-related genes from the literature (Li et al., 2022; Tsvetkov et al., 2022). A protein-protein interaction network was created using the STRING database to further explore relationships among these genes. We identified 128 ferroptosis-related genes to be closely associated with cuproptosis-related genes. Therefore, we constructed a novel signature (CRF) by combing cuproptosis-related genes with ferroptosis-related genes. The protein-protein interaction network was constructed based on the STRING online database and visualized using Cytoscape software (Figure 5A). By taking the intersection of the DEGs, WGCNA-based hub genes and CRF genes, three overlapping genes (POR, SLC7A5, and STAT3) were identified for SIC (Figure 5B). The diagnostic values of the three genes were confirmed by ROC curve and AUC analysis. As shown in Figure 5C, the AUC values of POR, SLC7A5 and STAT3 produced diagnosis powers for SIC of 0.922727, 0.990909, and 0.963636, respectively. Subsequent GSEA showed that the hallmark gene sets of the three genes were for fatty acid metabolism (Figures 6A–C).

### Construction of a TF-miRNA-hub gene network for SIC

**FIGURE 7:**
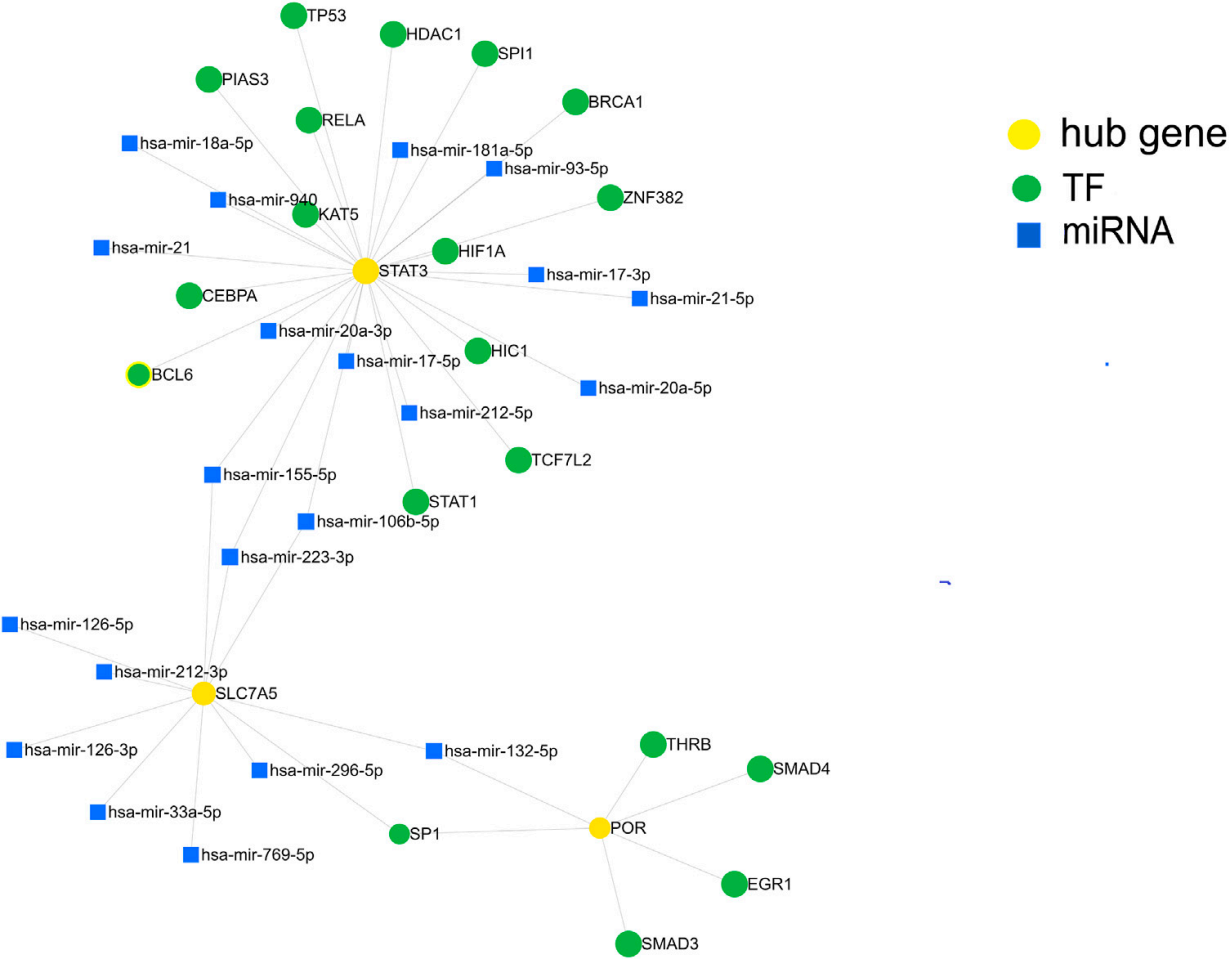
Construction of the TF-miRNA-hub gene network in sepsis-induced cardiomyopathy based on miRnet.

We further investigated the regulatory mechanism of these three genes in SIC. The target miRNAs and TFs of the three genes were identified and then the TF–miRNA-hub gene network was constructed based on miRnet. Finally, a TF–miRNA-hub gene network, which included the three genes, 19 TFs, and 21 miRNAs, was constructed with 45 edges (Figure 7).

### Screening for SIC therapeutic drugs

Potential therapeutic compounds for SIC associated with the three hub genes were screened for using DGIdb (Table 1). We identified 15 drugs as potential therapeutic compounds for SIC.

**TABLE 1:** The potential compounds of three genes were identified using DGIdb.

Gene	Drug	match_type	Sources
POR	NICOTINE	Definite	PharmGKB
POR	MIDAZOLAM	Definite	PharmGKB
POR	CYCLOSPORINE	Definite	PharmGKB
POR	ZIDOVUDINE	Definite	PharmGKB
POR	SIROLIMUS	Definite	PharmGKB
POR	ATORVASTATIN	Definite	PharmGKB
POR	SUNITINIB	Definite	PharmGKB
POR	TACROLIMUS	Definite	PharmGKB
SLC7A5	MELPHALAN	Definite	PharmGKB
STAT3	ACITRETIN	Definite	TTD
STAT3	PYRIMETHAMINE	Definite	DTC
STAT3	DIGITOXIN	Definite	DTC
STAT3	NICLOSAMIDE	Definite	DTC
STAT3	DIGOXIN	Definite	DTC
STAT3	OUABAIN	Definite	DTC

## Discussion

Sepsis has become one of the top ten causes of death in both developed and developing countries (Reinhart et al., 2017). Sepsis-induced cardiomyopathy, which is common and closely associated with higher mortality, has been the focus of attention. Although intensive efforts have been made to understand the molecular mechanism of sepsis-induced cardiomyopathy, a precise definition and prognostic parameters remain uncertain. Although biomarkers were added to the physiological parameters of sepsis-induced cardiomyopathy, their release was observed to be generally inconsistent with the severity of the disease (Hollenberg and Singer, 2021).

Programmed cell death is critical for organ development, tissue homeostasis, as well as the prevention of tissue injury and tumorigenesis (Fuchs and Hermann, 2011). As a newly recognized form of programmed cell death, ferroptosis is closely related to the pathogenesis of a large variety of diseases, such as cancer (Lei et al., 2022), cardiovascular disease (Fang et al., 2019), Parkinson’s disease (Bruce et al., 2016), chronic obstructive pulmonary disease (Yoshida et al., 2019), and autoimmune hepatitis (Zhu et al., 2021). Recent studies have shown that ferroptosis is closely related to the occurrence of sepsis and plays a crucial role in sepsis organ damage (Liu et al., 2022a). Vital roles of ferroptosis in the pathogenesis of SIC were also identified and ferritinophagy-mediated ferroptosis is involved in sepsis-induced cardiac injury (Ning et al., 2020). Both ferroptosis and cuproptosis are associated with mitochondria and are involved in the progression of a number of malignant tumors. Cuproptosis is also closely related to cardiovascular diseases. In this study, we screened cuproptosis- and ferroptosis-related genes using a bioinformatics approach. Previous studies have only focused on WCGNA to identify key genes; however, we applied text mining and WCGNA and, thereby, identified three hub genes (POR, SLC7A5, and STAT3). The diagnostic values of the three genes for SIC were confirmed using ROC curves.

The three hub genes are all associated with ferroptosis, their connection with cuproptosis in the pathophysiology of SIC is unknown. Cytochrome p450 oxidoreductase (POR) encodes an oxidoreductase that is indispensable for metabolism (Sugishima et al., 2019). The reactive oxygen species (ROS) that initiate ferroptosis come from a variety of sources, including iron-mediated Fenton reactions, mitochondrial ROS, and membrane-associated ROS driven by the NOX protein family. Polyunsaturated fatty acid-containing phospholipids are the main substrates of lipid peroxidation in ferroptosis, which is positively regulated by POR (Liu et al., 2022b). In a recent bioinformatics analysis of sepsis-induced cardiomyopathy, POR was selected as the central gene and its expression level was higher than that of the control group. GSEA then demonstrated POR to have a close relationship with cardiac metabolism, necroptosis and apoptosis of cells in SIC (Li et al., 2021). Solute carrier family 7 member 5 (SLC7A5), also known as L-type amino acid transporter (LAT1) (Galluccio et al., 2013), is a sodium-independent high-affinity amino acid transporter. SLC7A5 together with SLC3A2 mediate cellular uptake of the large neutral amino acids, phenylalanine, tyrosine, leucine, and tryptophan (Mastroberardino et al., 1998). SLC7A5 may affect the development of many diseases by regulating ferroptosis (Mao and Ma, 2022). Signal Transducer and Activator of Transcription 3 (STAT3) is a member of the STAT protein family. It can trigger transcription of a variety of genes in response to cytokines, which play a key role in many cellular processes, such as cell growth, apoptosis and ferroptosis. Accumulating evidence indicates STAT3 to be a converging point of multiple inflammatory response pathways in sepsis pathophysiology (Lei et al., 2021). This indicates that these genes are promising targets for drug development. Regulating fatty acid metabolism can sensitize cells to ferroptosis. In our study, GSEA showed that the hallmark gene sets of the three genes were for fatty acid metabolism. Therefore, we speculate that fatty acid metabolism maybe also involved in cuproptosis in SIC patients, but further experiments are needed to confirm this.

In addition to single protein-expressing genes, whole pathway networks may be deregulated in SIC. This may be mediated by miRNAs. miRNAs are associated with the pathophysiological process of many diseases (Formosa et al., 2022) and are involved in the occurrence and development of SIC (Beltrán-García et al., 2021). We therefore built a TF-miRNA-hub gene network depending on the shared dataset and published literature. We identified 19 TFs and 21 miRNAs as the master regulators of the resulting gene regulatory network that have the largest connectivity with the three co-expressed genes associated with SIC. Finally, by screening DGIdb, target therapeutic compounds for the hub genes were identified.

There are some limitations to this study. We only extracted data from databases and did not validate these data with animal experiments or clinical specimens. The screening results of this study were relatively accurate, which provides theoretical support for clinical drug development.

## Conclusion

In summary, this study used bioinformatics methods to identify hub genes and pathways involved in sepsis-induced cardiomyopathy and revealed the potential role of ferroptosis and cuproptosis. Our findings indicated 15 drugs as candidates for sepsis-induced cardiomyopathy therapy. Further studies are needed to explore the causal relationship between ferroptosis and cuproptosis and sepsis-induced cardiomyopathy and to provide prognostic markers. Overall, our analysis provides a workflow for predicting biomarkers and drug targets, which can be widely used in other diseases.

## Data Availability

The datasets presented in this study can be found in online repositories. The names of the repository/repositories and accession number(s) can be found in the article/supplementary material.
